# Application of Half-Transected and Self-Pulling Esophagojejunostomy in Total Laparoscopic Gastrectomy for Gastric Cancer: A Safe and Feasible Technique

**DOI:** 10.1155/2022/2422274

**Published:** 2022-06-13

**Authors:** Hongtao Wan, Jianyong Xiong, Yanglin Chen, Haiyun Wei, Ren Tang, Chao Chen, Qing Sun, Jing Xu, Bo Yi

**Affiliations:** Jiangxi Province Cancer Hospital, Nanchang, Jiangxi, China

## Abstract

**Objective:**

This study introduces a technique for esophagojejunostomy with half transected and self-pulling (HTSP) and evaluates the safety, feasibility, and clinical results of this technique in totally laparoscopic total gastrectomy (TLTG).

**Materials and Methods:**

From May 2019 to March 2021, 42 patients (HTSP group) who underwent HTSP-TLTG surgery in the Department of Abdominal Tumor Surgery of Jiangxi Cancer Hospital were included in this study. The control group consisted of 50 patients undergoing conventional TLTG surgery (conventional anastomosis group) performed by the same surgical team from March 2018 to March 2020. The clinical data of the two groups were retrospectively analyzed and compared.

**Results:**

The mean operation time of the HTSP-TLTG surgery was 166.7 ± 13.1 minutes and the anastomosis time was 20.8 ± 2.0 minutes, which were significantly shorter than those of traditional TLTG (*P* < 0.05). There were no significant differences between the two groups in blood loss, time to first exhaust, postoperative hospital stay, and incidence of surgery-related complications.

**Conclusion:**

HTSP is a safe and feasible way of endoscopic esophagojejunal anastomosis, which requires a relatively low suture technique under endoscopy, and is suitable for promotion.

## 1. Introduction

Laparoscopic treatment of gastric cancer has been widely carried out in many countries around the world [1–3]. With the continuous maturity and improvement of laparoscopic technology, it is not limited to be the treatment for early gastric cancer, and its efficacy in advanced gastric cancer has gradually been recognized [4–8]. Compared with laparoscopic-assisted radical gastrectomy (LAG), total laparoscopic gastrectomy (TLG) has the advantages of better visualization, shorter operation time, less postoperative pain, and smaller incision, so it is widely accepted and respected by surgeons [9–11]. In view of the difficulty and high technical requirements of total esophagojejunostomy, TLTG is not as widely used as total laparoscopic distal gastrectomy (TLDG) [12–15].

Currently, esophagojejunostomy is mainly performed with circular and linear staplers. The former mainly includes the peroral stapler anvil device (OrVil™) method, reverse puncture placement (RPD) method, purse-string stapler method, and manual suture method; the latter mainly includes functional end-to-end anastomosis (FETE), partially overlapping side-to-side anastomosis (overlap group), and “*π*-type” anastomosis [16–20]. Anastomosis techniques are continuously improving and innovating but still face many problems, such as difficulty in laparoscopic anvil implantation, difficulty in common opening suturing, and high price.

To address these issues, our team created the half-transected self-pulling (HTSP) esophagojejunostomy technique in May 2019, based on a summary of traditional surgical experience, combined with the advantages of linear staplers. In this report, we will describe the novel stapling technique in detail and analyze its feasibility and short-term safety by comparing its clinical results with conventional TLTG (overlap or functional end-to-end anastomosis (FETE)). Our preliminary experiments have shown that HTSP is a simple and safe way for endoluminal esophagojejunostomy. Without the need to add any surgical steps, this technique makes the manual suture link of the common opening simpler and faster, further reduces the difficulties, and shortens the anastomosis time; therefore, it is more easily accepted and popularized by surgeons.

## 2. Material and Methods

Between May 2019 and March 2021, 42 patients (HTSP group) who underwent HTSP-TLTG surgery in the Department of Abdominal Tumor Surgery of Jiangxi Cancer Hospital were included in this study. The control group consisted of 50 patients (conventional anastomosis group) undergoing conventional TLTG surgery performed by the same surgical team from March 2018 to March 2020. Preoperative evaluation methods mainly include endoscopy, ultrasonography, and enhanced CT.

Inclusion criteria of this study were as follows: (1) preoperative pathological confirmation of gastric adenocarcinoma; (2) endoscopy confirmed that the tumor is located in the gastric body, fundus, or cardia; (3) preoperative CT staging is cT1-4aN0-2M0; (4) patients signed an informed consent form; (5) approval by the Ethics Committee of Jiangxi Cancer Hospital. Patient information was collected, including age, gender, body mass index (BMI), operation and anastomosis time, blood loss, pathological stage, and postoperative complications.

### 2.1. Surgical Approach for HTSP-TLTG

**Figure 1:**
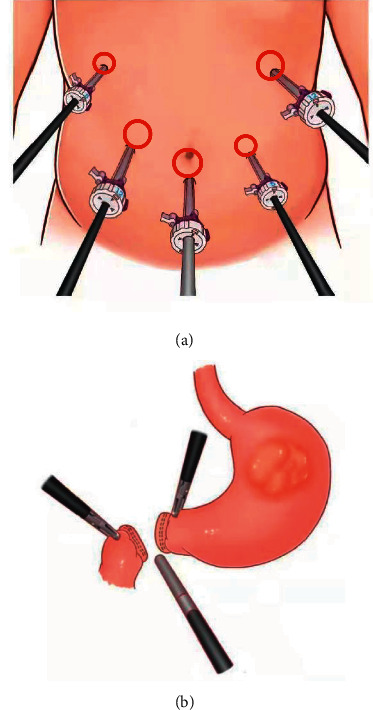
(a) Trocar hole position distribution, 1 cm below the umbilical disposal into the 10 mm trocar as the observation hole. (b) The duodenum after transection.

Gastric tubes and urinary catheters were routinely placed in all patients before surgery. Under general anesthesia with endotracheal intubation, the patient was placed in the reverse Trendelenburg position with legs apart and the head elevated to about 15^。^. The chief surgeon was on the left side of the patient, the assistant was on the right side, and the camera holder was between the legs. The five-hole method was used in the operation. A longitudinal incision of 10 mm with trocar was made 1 cm below the umbilicus as the observation hole, and it was also used to establish pneumoperitoneum and maintain CO_2_ pressure at 12–14 mmHg (1 mmHg = 0.133 kPa). A 12 mm trocar was placed 2 cm below the costal margin of the left anterior axillary line and 2 cm above the umbilicus of the right midclavicular line as the main operating hole for the chief surgeon and the right-hand operating hole for the assistant, respectively. A 5 mm trocar was placed 2 cm below the costal margin of the right anterior axillary line and 2 cm above the umbilicus of the left midclavicular line as the left-hand operating hole for the chief surgeon and assistant, respectively (Figure 1(a)). The abdomen and pelvis were explored to rule out peritoneal implants and distant metastasis. Routine D2 dissection was completed according to the standardized requirements for radical gastrectomy for gastric cancer. The need to transect part of the diaphragmatic crus was decided by judging the level of tumor location, and the duodenum was transected with a linear stapler (Figure 1(b))

### 2.2. Reconstruction of the Alimentary Canal with HTSP-TLTG

**Figure 2:**
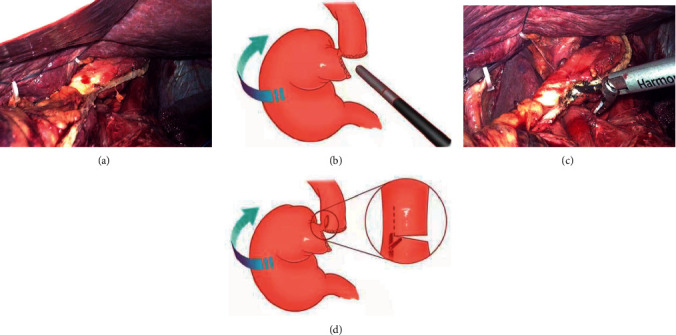
(a, b) The linear stapler transected the lower esophagus segment. (c, d) Ultrasonic knife perpendicular to the esophageal tangent to take a longitudinal hole, with the position under the action of gravity to produce downward-to-right traction.

**Figure 3:**
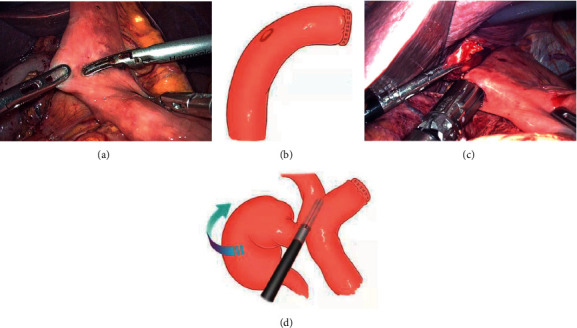
(a, b) The intestinal wall was drilled 8 cm away from the stump. (c, d) Jejunum was lifted and a linear stapler was used to perform side-to-side anastomosis between the posterior wall of the esophagus and the opposite limbus of the jejunum.

**Figure 4:**
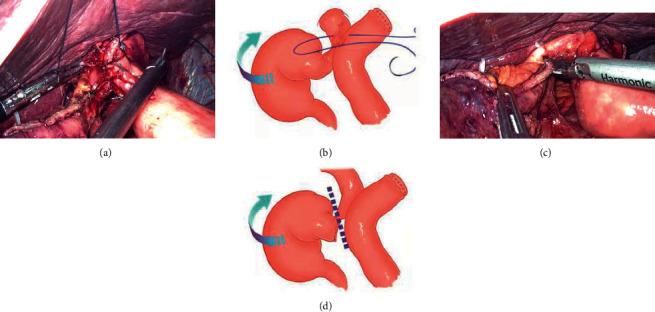
(a, b) A 3-0 barbed wire from left to right continuous sutured closure common opening. (c, d) Ultrasound knife transected the remaining part of the esophagus.

**Figure 5:**
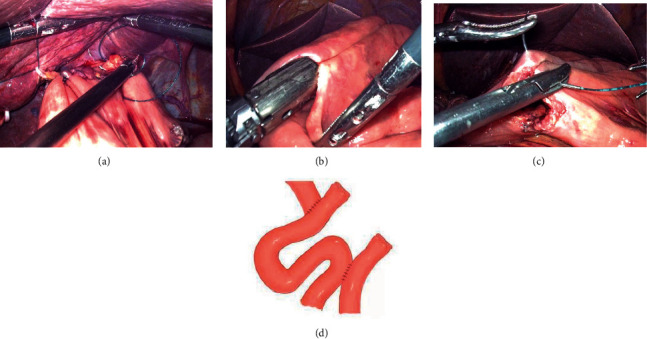
(a) 3-0 barbed wire from right to left for jejunal seromuscular-diaphragmatic suture. (b) The proximal jejunum was anastomosed to the jejunum side at 40 cm away from the esophagojejunostomy. (c, d) 3-0 barbed wire sutured to close the common opening.

The lower esophagus is partially dissected (the gastric corpus tumor was cut off from the cardia and the esophagogastric junction tumor was cut off from the upper edge of the tumor) by using a linear stapler (Figures 2(a) and 2(b)), a small longitudinal hole perpendicular to the tangential line of the esophagus was made with an ultrasonic knife; then, the stomach and the greater omentum are all sent to the right abdomen of the patient, and the traction force to the right and downward of the esophagus is formed under the action of gravity, which is called “half-transected self-pulling” (Figures 2(c) and 2(d)). The jejunum was dissected with a linear stapler at 15-20 cm from the Treitz ligament, and a hole was made in the distal jejunum wall opposite to the mesentery at a distance of 8 cm from the cut end (Figures 3(a) and 3(b)). After that, the chief surgeon and the assistant switched positions, with the chief surgeon on the right side of the patient. The jejunum was lifted up, and the linear stapler was used to tilt 45° to complete the lateral-lateral anastomosis between the posterior esophageal wall and the jejunum wall opposite to the mesentery (if the tumor is low, the anastomosis is completed outside the crus of the diaphragm, and if the tumor is high, the anastomosis is completed inside the crus of the diaphragm) (Figures 3(c) and 3(d)). The common opening was closed with 3-0 barbed sutures from left to right in a continuous manner (Figures 4(a) and 4(b)). After the sutures were closed to about 3/4, the assistant cut off the remaining part of the esophagus with an ultrasonic knife (Figures 4(c) and 4(d)). After the common opening was completely closed, the jejunal seromuscular-diaphragmatic suture was continued from right to left to reinforce the common opening (Figure 5(a)). The proximal jejunum was perforated at 8 cm from the break, and the proximal jejunum was laterally anastomosed with the jejunum at about 40 cm from the esophagojejunostomy (Figure 5(b)), 3-0 barbed line full-thickness suture from left to right to close the common opening, followed by reinforcement of the seromuscular layer from right to left (Figures 5(c) and 5(d)). The specimen was placed in a specimen bag and removed through an extended umbilical incision and placed on a negative pressure drainage tube in the splenic fossa.

### 2.3. Evaluation Criteria

The surgical indicators, the occurrence of postoperative complications, and the postoperative recovery between the two groups were compared. The surgical indexes included operative time, anastomosis time, and intraoperative blood loss. The postoperative recovery indexes included the time to the first postoperative exhaust and the postoperative hospital stay. Postoperative complications included abdominal or anastomotic bleeding, anastomotic leakage, anastomotic stricture, pancreatic leak, lymphatic leak, abdominal infection, pulmonary infection, and reflux esophagitis.

### 2.4. Postoperative Management

**Figure 6:**
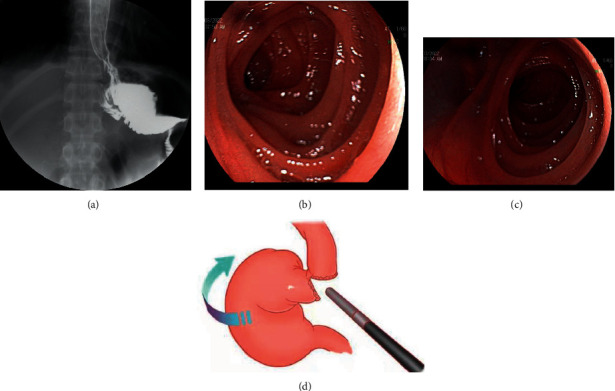
(a) Angiography on day five after surgery showing no anastomotic leakage. (b) Electronic gastroscopy 6 months after surgery showing no anastomotic stenosis. (c) Inside the dotted circle is the upper margin requiring a pathological examination.

The gastric tube was removed on the first day after surgery, and the patient was allowed to ingest a small amount of liquid food several times after the first postoperative exhaust. After operation, the abdominal cavity or anastomotic bleeding, lymphatic leakage, and pancreatic leakage were determined by the drainage of the abdominal cavity drainage tube. An upper gastrointestinal X-ray was performed on postoperative day 5 to evaluate for anastomotic leakage (Figure 6(a)). On the 6th day after operation, CT examination was performed to check for the presence of lung and abdominal infection. The patient was discharged 8–10 days postoperatively. Electronic gastroscopy was performed 6 months after discharge to check for the presence of anastomotic stenosis (Figures 6(b) and 6(c)).

### 2.5. Statistical Methods

SPSS 22.0 was used to analyze the data. Normally distributed measures were expressed as mean ± standard deviation and the *t*-test was used for the two samples; non-normally distributed measures were expressed as median (range) and the Mann-Whitney *U* test was used. *P* < 0.05 was considered a statistically significant difference.

## 3. Results

### 3.1. Comparison of Patient Characteristics

The general information of the patients in the two groups was compared (Table 1). There were 42 patients in the HTSP group, including 33 men and 9 women, with a median age of 63.0 years (17–80 years) and a mean BMI of 21.4 ± 2.3 kg/m^2^. The average diameter of the tumor was 21.4 ± 2.3 cm, and 19 of them were located in the fundus or upper stomach, while 23 were located in the middle of the stomach. There were 50 patients in the overlap or FETE group, including 40 men and 10 women, with a median age of 61.5 years (38–83 years) and a mean BMI of 21.2 ± 2.2 kg/m^2^. The average diameter of the tumor was 3.02 ± 1.8 cm, and 21 of them were located in the fundus or upper stomach, while 29 were located in the middle of the stomach.

### 3.2. Comparison of Intraoperative and Postoperative Conditions

The operation was successfully completed in both groups. There was no conversion to laparotomy due to anastomotic problems and no positive margin in both groups. There was no significant difference in blood loss, first exhaust time, and postoperative hospital stay between the two groups. However, the operation time and anastomosis time of the HTSP group were shorter than those of the traditional anastomosis group, and the differences were statistically significant (all *P* < 0.05) (Table 2).

### 3.3. Comparison of Surgery-Related Complications

Intraoperative complications, including spleen injury and vascular bleeding, were not present in this study in either group. The incidence of postoperative complications in the HTSP group was 4.7% (2/42), including 1 case of lymphatic leakage (3 days after surgery) and 1 case of pulmonary infection (4 days after surgery). All the patients were cured after conservative treatment, and there was no significant difference compared with the conventional TLTG group. Other common complications such as anastomotic or duodenal stump leakage, anastomotic bleeding, and intestinal obstruction or internal hernia were not found in this study. During the follow-up period, no HTSP patient complained of reflux symptoms or anastomotic stenosis, and only one patient had liver metastasis 9 months after operation (Table 3).

## 4. Discussion

In recent years, although many studies have confirmed the safety and feasibility of TLTG with different anastomosis methods, TLTG has not been carried out as widely as TLDG [12–15]. The main reason is that the esophageal-jejunal anastomosis is difficult, with high technical requirements and high surgical risks. Compared with the circular stapler, the linear stapler is easier to operate, which is less likely to cause anastomotic stenosis, and is suitable for the esophagus with the small lumen. Therefore, it is widely recognized as an anastomosis method in many current methods [20–25]. However, the current mainstream linear anastomosis methods overlap and FETE have some shortcomings [14, 26]as follows: (1) In terms of closing common openings, overlap and FETE cannot provide a stable suture field of view, resulting in manual suture difficulties and high technical requirements for the surgeon. (2) Both the two methods will retract into the posterior mediastinum after esophageal transection, resulting in difficult operation and is unsuitable for cases requiring high esophageal transection.

Our team has tried and improved various TLTG methods since February 2018 and found that “half-transect” of the esophagus can avoid esophageal retraction into the posterior mediastinum and reduce the difficulty of surgery. Then, the other method was with the change of posture and full use of the gravity of the stomach and the greater omentum to produce downward and right traction on the esophagus, so as to close the common opening to provide a stable suture vision, called “self-pulling.” Combining these two methods to form a “half-transected self-pulling modified overlap anastomosis” makes up for the above-mentioned deficiencies of linear anastomosis without adding any surgical steps and financial burden on the patient. Based on these technical features, we name it “half-transected self-pulling, HTSP.”

The operation skills of HTSP are as follows: (1) The lower segment of the half-transected esophagus should be dismembered about 3/4. Excessive dismemberment is not conducive to revealing the true esophageal cavity, which may lead to entry into the submucous tract during anastomosis. However, too little dismemberment will lead to a huge common opening and increase the suture time. During operation, the chief surgeon is located on the left side of the patient, and the termination line of the linear stapler just falls on the right edge of the esophagus. (2) Before closing the common opening, the patient's head is lifted and the foot is lowered and tilted to the right. Through the adjustment of posture, the appropriate self-pulling force of the esophagus can be given, so as to provide a clear and stable suture vision for the surgeon. After different attempts on the position of the surgeon during suture, we believe that the suture with the chief surgeon on the right side of the patient and common openings is an ideal position. (3) To close the common opening by a continuous full-thickness suture from left to right with 3-0 barbed wire, the traditional control group was reinforced with the esophageal-jejunal seromuscular layer from right to left, while the team was reinforced with the diaphragmatic-jejunal seromuscular layer from right to left. This reinforcement method does not increase the risk of intraoperative complications and can reduce the risk of anastomotic leakage by reducing the tension of the anastomotic stoma.

The results of this study showed that without any increase in surgical steps, surgery-related complications, and economic burdens, the operation time was shortened to about 158 minutes on average, and the reconstruction time was shortened to 21 minutes on average. No serious complications occurred, which were attributed to the stable suture vision provided by this anastomosis method, thereby reducing the technical difficulty of the suture. The HTSP method has the following advantages: (1) HTSP can not only provide a stable and clear suture vision, but also provide better surgical field exposure and reduce the secondary tissue damage caused by a repeated grasp of the esophagus and jejunum during operation. (2) The downward-to-right traction of the esophagus by the HTSP method can be suitable for cases requiring higher level (gastroesophageal junction tumor) and improves the resection rate of R0. (3) The HTSP methods for laparoscopic suture technology requirements are relatively low, the learning curve is relatively short, and is more suitable for promotion. HTSP also has some shortcomings, that is, the specimens cannot be completely severed before anastomosis, so it is impossible to perform a rapid frozen pathological examination of the esophageal margin, and there is a potential risk of positive margin. For this reason, our team has also made different attempts to determine the upper incision margin. At present, for patients who need a rapid frozen pathological examination to determine the upper margin, our primary approach is to laparoscopically clip the partial proximal esophageal margin after half-transection and perform a rapid frozen pathological examination (Figure 6(d)). Of course, we will continue to explore a simpler and more effective method for determining the upper margin.

In summary, the HTSP method is a simple, safe, and feasible laparoscopic esophagojejunostomy technique, which can reduce the technical requirements for surgeons and has an important reference value for the extensive development of TLTG in the future. However, this study is only a single-center small-sample study, and comparative studies with multiple sample sizes, prospective randomized controlled trials, and long-term follow-up results are needed to further confirm the efficacy of this method.

## Figures and Tables

**Table 1:** Comparison of general data of gastric cancer patients in the half-transected and self-pulling group (HTSP group) and the traditional anastomosis group (overlap or FETE group).

Groups	Number	Age (years, medain (range))	Male (number (%))	BMI (kg/m^2^,±s）	Tumor location (%)				
					Upper part		Middle part		
HTSP	42	63.0 (17∼80)	33 (78.5)	21.4 ± 2.3	19 (45.2)		23 (54.7)		
Overlap or FETE	50	61.5 (38∼83)	40 (80.0)	21.2 ± 2.2	21 (42.0)		29 (58.0)		
Statistic value		*U* = 930.0	*X* ^2^ = 0.168	*t* = 0.478	*X* ^2^ = 0.097				
*P* value		0.349	0.866	0.643	0.755				

Groups	Tumor size (cm)	Clinical staging (number (%))				T staging (number (%))			
		I	II	IIIA-B	IIIA-B	T1	T2	T3	T4
HTSP	2.74 ± 1.6	8 (19.0)	12 (28.6)	19 (45.2)	3 (7.1)	4 (9.5)	6 (14.2)	5 (11.9)	27 (64.3)
Overlap or FETE	3.02 ± 1.8	9 (18.0)	14 (28.0)	22 (44.0)	5 (10.0)	7 (14.0)	4 (8.0)	2 (4.0)	37 (74.0)
*P* value	0.428	0.971				0.334			
Groups	N staging (number (%))				Pathological staging (number (%))				
	N0	N1	N2	N3	I	II	IIIA-B	IIIC	
HTSP	18 (42.9)	6 (14.3)	9 (21.4)	9 (21.4)	10 (23.8)	8 (19.0)	16 (38.1)	8 (19.0)	
Overlap or FETE	17 (34.0)	6 (12.0)	6 (12.0)	21 (42.0)	8 (16.0)	9(18.0)	13 (26.0)	20 (40.0)	
*P* value	0.190				0.166				

**Table 2:** Comparison of intraoperative and postoperative conditions between half-transected and self-pulling group and the traditional anastomosis group.

Groups	Number	Operation	Anastomosis duration (min)	Blood loss	Time to first exhaust	Postoperative
HTSP overlap or FETE	42 50	166.7 ± 13.1 181.9 ± 13.2	20.8 ± 2.0 29.9 ± 1.7	72.1 ± 23.6 73.5 ± 28.1	64.7 ± 18.7 65.4 ± 17.9	7.4 ± 1.6 7.5 ± 1.9
*T* value	5.520	24.02	0.257	0.191	0.148	0.148
*P* value	0.000	0.000	0.798	0.798	0.849	0.882

**Table 3:** Comparison of postoperative complications between half-transected and self-pulling group and traditional anastomosis group in patients with gastric cancer [number (%)]

Characteristic	HTSP (*n* = 42)	Overlap or FETE (*n* = 50)	*P* value
Postoperative complication (%)	4.7	4.0	0.858
Intra-peritoneal or digestive tract hemorrhage (*n*)	0	0	
Anastomotic leakage(*n*)	0	0	
Anastomotic stenosis (*n*)	0	0	
Pancreatic leakage(*n*)	0	1	
Lymphatic leakage (*n*)	1	0	
Intra-abdominal infection or abscess (*n*)	0	0	
Pulmonary infection (*n*)	1	1	
Reflux esophagitis (*n*)	0	0	

## Data Availability

The research data used to support the findings of this study are included within the article.
